# A progressive and complex clinical course in two family members with *ERF*-related craniosynostosis: a case report

**DOI:** 10.1186/s12881-020-01015-z

**Published:** 2020-05-05

**Authors:** Izabella Körberg, Daniel Nowinski, Marie-Louise Bondeson, Malin Melin, Lars Kölby, Eva-Lena Stattin

**Affiliations:** 1 Department of Immunology, Genetics and Pathology, Uppsala University, Science for Life Laboratory, Uppsala, Sweden; 2 Department of Surgical Sciences, Plastic Surgery, Uppsala University, Uppsala, Sweden; 3 Department of Plastic Surgery, Institute for Clinical Sciences, Sahlgrenska Academy, University of Gothenburg, Sahlgrenska University Hospital, Gothenburg, Sweden

**Keywords:** *ERF*, Craniosynostosis, Intracranial hypertension

## Abstract

**Background:**

ERF-related craniosynostosis are a rare, complex, premature trisutural fusion associated with a broad spectrum of clinical features and heterogeneous aetiology. Here we describe two cases with the same pathogenic variant and a detailed description of their clinical course.

**Case presentation:**

Two subjects; a boy with a BLSS requiring repeated skull expansions and his mother who had been operated once for sagittal synostosis. Both developed intracranial hypertension at some point during the course, which was for both verified by formal invasive intracranial pressure monitoring. Exome sequencing revealed a pathogenic truncating frame shift variant in the *ERF* gene.

**Conclusions:**

Here we describe a boy and his mother with different craniosynostosis patterns, but both with verified intracranial hypertension and heterozygosity for a truncating variant of *ERF* c.1201_1202delAA (p.Lys401Glufs*10). Our work provides supplementary evidence in support of previous phenotypic descriptions of *ERF*-related craniosynostosis, particularly late presentation, an evolving synostotic pattern and variable expressivity even among affected family members.

## Background

Craniosynostosis (CS) is clinically and genetically a heterogenous congenital anomaly with an incidence of 1 in 2500 live births [1]. In most patients, CS presents as an isolated anomaly (70–75%; non-syndromic). In 25–30% of all patients, CS is manifested with additional anomalies and/or developmental delay (syndromic).

The most common clinical feature in CS is abnormal head shape. This is a consequence of growth of the underlying brain and restriction of skull growth due to premature ossification of skull sutures [1]. CS can result in numerous morphologic and functional abnormalities, including craniofacial malformation, increased intracranial pressure and intellectual disability. It is usually classified based on suture fusion type: sagittal, metopic, bi−/unicoronal, bi−/unilambdoid and complex, or multisutural.

Bilateral lambdoid and sagittal synostosis (BLSS) was first described 1976 by Neuhauser et al,. as a syndrome called “craniofacial dyssynostosis”, [OMIM 218350]. Seven patients, including two siblings, presenting with BLSS where described, which also presented with short stature, Chiari type 1, intellectual disability and suspected autosomal recessive inheritance [2]. BLSS was later called “Mercedes Benz pattern” of CS due to the characteristic symbol of the three fused sutures on CT imaging [3]. This is a rare complex trisuture synostosis with a distinct clinical picture, including severe frontal bossing, biparietal narrowing, turribrachycephaly, occipital bulging or flatness, and low set ears [4]. Chiari I malformation is common and may require neurosurgical decompression [5].

The aetiology of CS is heterogeneous, and several chromosomal hotspot loci and candidate genes have been associated with CS [6–8]. One of the most recent addition is ETS2 repressor factor (*ERF*), in which pathogenic variants have been identified in patients presenting with CS [9–12].

Here we describe the clinical and molecular aspects of CS in a patient and his mother with a pathogenic variant in the *ERF* gene.

## Case presentation

### Clinical genetic investigation

All patients with craniosynostosis and craniofacial syndromes, referred to the Craniofacial centre in Uppsala, are assessed by a team clinical geneticist and formally included in genetic research with approval from the ethical committee (Dnr 2013/294). The Craniofacial team in Uppsala is one of two national reference centres for paediatric craniofacial surgery and has a close collaboration with the other licensed team in Gothenburg. Data from the care of the patients is entered in a quality registry with 100% coverage ratio. Blood samples are obtained from the patients and parents in connection to surgery or outpatient visits.

Patients with coronal, complex or atypical craniosynostosis are routinely investigated for chromosomal abnormalities and/or genetic variants with numerous methods including microarray analysis and a craniosynostosis gene panel. Selected patients with negative findings on routine genetic work-up are investigated further with whole-exome sequencing.

### Subjects

A baby-boy was referred at 2 months of age to the Craniofacial Centre, Uppsala University Hospital, due to craniosynostosis. He was born after an uneventful pregnancy in gestational week 38 + 3. The birth weight was 2.586 kg, and the length 46 cm. He was the first child of non-consanguineous parents and had been hospitalized due to stagnation in weight gain. The mother had noticed an abnormal head shape of the boy and a computed tomography (CT) performed at the local hospital confirmed complete fusion of the sagittal suture and bilateral partial fusions of both lambdoid sutures.

**Fig. 1:**
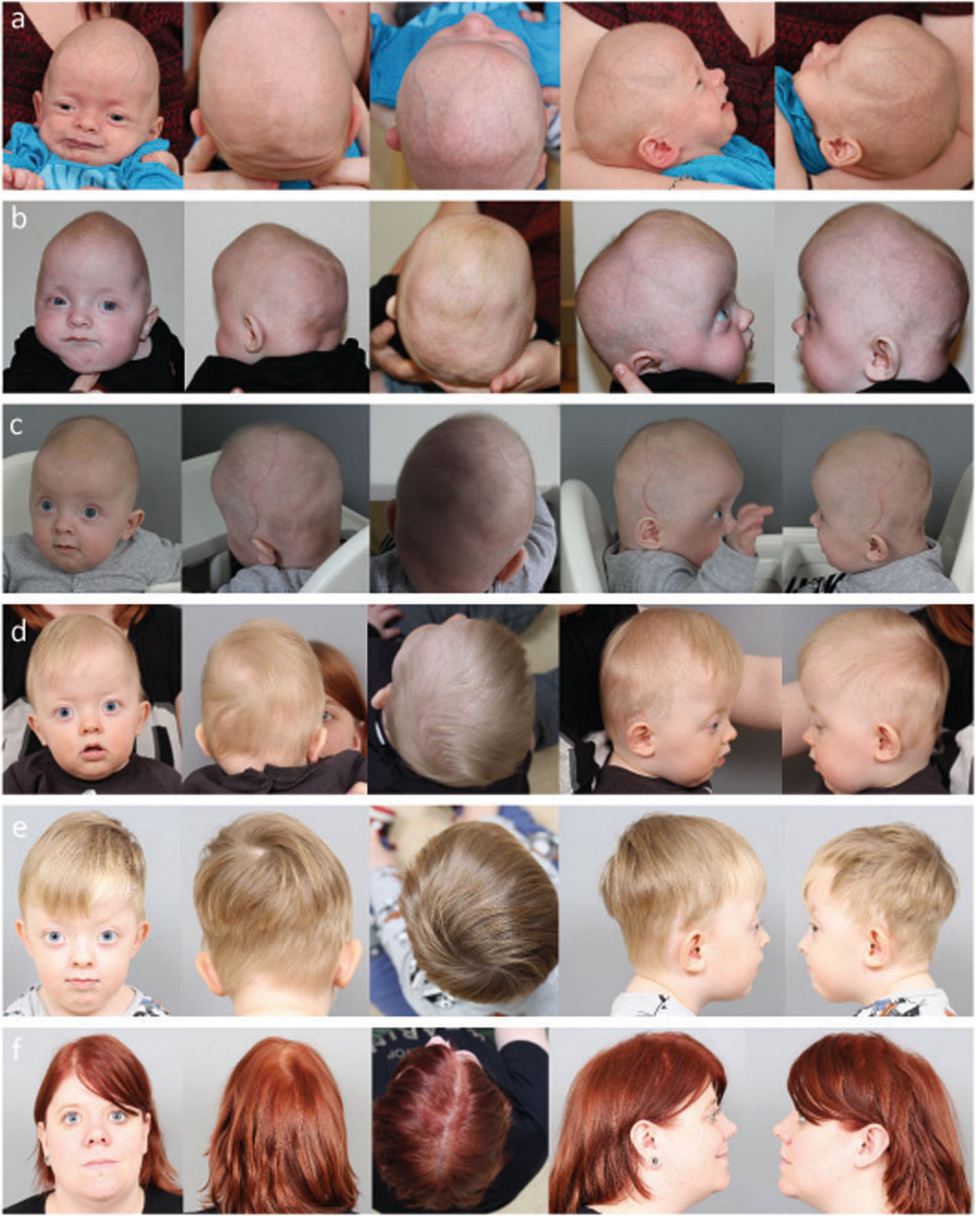
Craniofacial features of the index patient presenting with bilateral lambdoid and sagittal synostosis (**a**-**e**). Mother of the index patient (**f**). (**a**) First visit at the age of seven weeks and two days. Constriction of the posterior skull, with a compensatory forehead expansion, and slight hypertelorism (**b**) The forehead expansion was more pronounced before operation at the age of 5 months. (**c**) After operation at the age of 6 months. Note brachycephalic head shape, and mild exorbitism (**d**) After operation at the age of 13 months. Note brachycephalic head shape, and more pronounced exorbitism (**e**) Index patient at the age of 3 years and 6 months. Note a normal head shape, and even more pronounced exorbitism (**f**) Mother of the index patient at the age of 26 years, operated for a sagittal synostosis at the age of 5 years. Note a scaphocephalic head shape, and exorbitism

Clinically, there was a constriction of the posterior skull, compensatory forehead expansion, slight hypertelorism and slightly increased tension on the anterior fontanelle (Fig. 1). There were no other obvious malformations and no papilledema on ophthalmological examination. Magnetic Resonance Imaging (MRI) was performed and there was no Chiari type malformation or other signs of intracranial pathology. A craniectomy procedure was performed at 4 months of age. During follow-up, at 1 year of age, a decline in head circumference was noted, the head shape turned brachycephalic, there was a mild exorbitism, and repeated ophthalmological examinations revealed papilledema (Fig. 1). A repeat MRI showed development of a Chiari type 1 malformation and CT showed pathological gyral impression and restenosis with complete pansynostosis. At this stage a formal invasive monitoring of intracranial pressure was performed with an intraparenchymatous catheter, confirming intracranial hypertension (30–40 mmHg), which in turn resulted in performing a secondary posterior calvarial expansion with internal distractors. The operation and postoperative care were performed with the intracranial pressure catheter in place and a return to normal intracranial pressure dynamics was seen during the first days after surgery. At 3 years follow-up the boy was in good health, papilledema had resolved and head shape was normal (Fig. 1). The family move to the south of Sweden and the patient was therefore referred to the craniofacial team in Gothenburg for further follow-up. At 3.5 years of age, headaches and papilledema reappeared. Intracranial pressure was high (above 40 mmHg), and a third transcranial skull expanding procedure was performed including frontal remodelling and expansion and posterior expansion with springs over osteotomy lines. The boy was identified as a patient with a positive family history, complex craniosynostosis and negative findings on, at that time, implemented genetic CS panel. This warranted further investigation with clinical WES.

The mother of the boy had been referred to the craniofacial team in Gothenburg at the age of 4 years due to exophthalmos, left sided blindness and abnormal head shape (Fig. 1). There was no family history of craniofacial malformations. She had intermittent nocturnal headache and vomiting indicating raised intracranial pressure. Imaging with CT and MRI showed shallow orbits, marked gyral impressions and increased CSF around optic nerves. The anterior part of the sagittal suture was closed prematurely. Ophthalmological examination revealed left sided optic nerve atrophy. A formal 48-h ICP monitoring with an epidural sensor indicated intracranial hypertension. She was operated at the age of five with frontoorbital remodelling and biparietal expansion. After the operation headaches resolved and previous attention, and concentration difficulties were improved.

### Whole-exome sequencing

Clinical whole-exome sequencing (WES) and analysis protocols were developed by the Clinical genomics facility, Uppsala and were adapted as a clinical WES test at Dept. of Clinical Genetics, Uppsala University Hospital, Sweden, as described by Gudmundsson and collegues, with minor modifications [13]. Venous blood was collected from the boy and his parents and DNA was extracted using an automated system (QuickGene, QIAGEN Inc.) according to standard protocols. Whole exome sequencing was performed using a trio-based approach (patient, mother and father). In brief, 250 ng genomic DNA was used for library preparation with Clinical Research Exome and Sure Select^QXT^ Target Enrichment System (Agilent Technologies, Santa Clara, CA, USA). Enriched DNA was sequenced on an Illumina HiSeq2500 with 100 bp paired-end reads. Alignment of raw data to (GRch37/UCSChg19) and variant calling was performed using the Bcbio Nextgen v 0.8.9 pipeline tool (https://github.com/chapmanb/bcbio-nextgen). Briefly, alignment was performed using BWA 0.7.12, variant calling using GATK haplotype caller (GATK framework 3.2.4, Genomie Analysis TK 3.2.2), and quality control parameters were calculated using FastQC 0.11.3, Picard HsMetrics 1.96 (broadinstitute.github.io/picard) and GATK Depth of Coverage (GATK framework 3.2.4, Genomie Analysis TK 3.2.2). For filtering of variants BENCHlab NGS (Agilent Technologics Inc.) was used and allelic variants identified were classified according to the American College of Medical Genetics and Genomics and the Association for Molecular Pathology [14]. The pathogenic variant *ERF* (NM_ 006494.3) c.1201_1202delAA (p.Lys401Glufs*10) was confirmed by Sanger sequencing of DNA from the trio. Primers and PCR conditions are available upon request. PCR products were sequenced in both forward and reverse directions using BigDye terminator v3.1 cycle sequencing kit followed by automated sequencing on 3500 XL Genetic Analyzer (Applied Biosystems, Foster City, CA). Sequencing data was analysed using SequencePilot v3.5.1 software (JSI Medical Systems, GmbH).

### Identification of a pathogenic variant c.1201_1202delAA (p.Lys401Glufs*10) in ERF

**Fig. 2:**
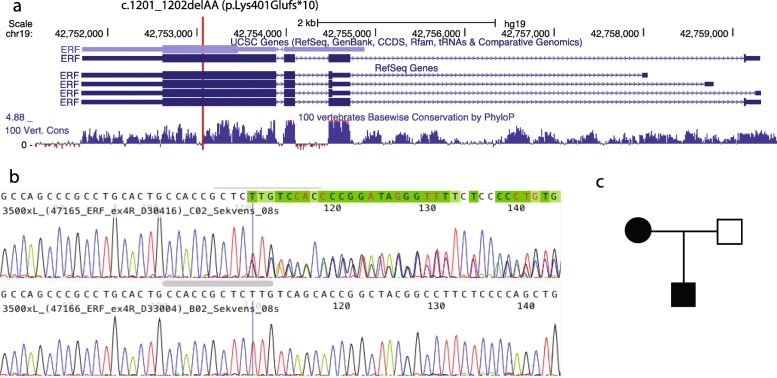
Structure of *ERF* gene and variant identified. (**a**) Main ERF transcript consist of 4 exons, *ERF* c.1201_1202delAA (p.Lys401Glufs*10) marked with red line. (**b**) Confirmation of truncating frameshift variant illustrated with sequencing. (**c**) pedigree

Whole-exome sequencing of the index patient (the boy) generated 95 M reads where 91% of the reads mapped to the reference genome. The average read depth was 94X and 97% of the exome was covered >10x. Filtering of variants revealed a heterozygous variant c.1201_1202delAA (p.Lys401Glufs*10) in *ERF* (NM_006494.3) (Fig. 2a). The variant was classified as likely pathogenic since it is predicted to result in a truncated protein; it is absent in 1000 genomes and has been reported in one out of 236,032 individuals in gnomAD. Sanger sequencing confirmed the presence of the c.1201_1202delAA (p.Lys401Glufs*10) variant in the index patient and his affected mother while the father was not a carrier (Fig. 2b,c).

## Discussion and conclusions

We report a boy and his mother carrying a heterozygous frameshift variant c.1201_1202delAA (p.Lys401Glufs*10), in *ERF* gene causing sagittal, and BLSS also called “Mercedes Benz pattern “of CS, and isolated sagittal CS respectively. Interestingly, both mother and son developed intracranial hypertension which was verified by invasive monitoring. Phenotypically there were several similarities, but also some differences. Both had sagittal synostosis, shallow orbits and mild hypertelorism (Fig. 1). Only the son, however, had lambdoid suture involvement, which probably explained why only he developed a Chiari 1 malformation.

An interesting observation is the pansynostosis (including coronal sutures) that developed over time in the index patient. ERF-related pansynostosis has been described in previously reported cases [9, 12]. Thus, the postoperative pansynostosis observed here could be a postnatal progression of the sutural fusion, similarly to what is seen in Crouzon syndrome. Alternatively, the coronal fusion could be a phenomenon secondary to the early craniectomy procedure. Indeed, it is well known that secondary, postsurgical coronal synostosis develops in a proportion of patients with non-syndromic sagittal synostosis [15, 16]. However, the progressive evolution of slight exorbitism in the index patient rather indicates a primary and progressive sutural fusion causing growth restriction of the anterior skull base and orbits. Secondary coronal synostosis after surgery for sagittal synostosis, which is believed to be caused by the loss of growth promoting forces across the coronal sutures after the decompressive widening of the skull, has not been seen to cause progressive supraorbital retrusion and exorbitism.

The *ERF* sequence variant has been reported previously in four patients (family 12, and patient 19, and 25), family 12 presenting with pansynostosis, hypertelorism, delayed development, poor attention span and problems with writing (Table 1) [9]. Furthermore, the patient presented with brachydactyly of hands and feet, broad halluces and dysplastic ears, thus syndromic CS [9]. Patient 19 presented with pansynostosis and Chari-1 malformation, he had orbital hypertelorism, malar hypoplasia, frontal bossing, long philtrum, high palate, low set ears, inverted nipples and clinodactyly [12].

**Table 1:** Overview of the clinical features of *ERF* variant c.1201_1202delAA p.Lys401Glufs*10 as reported by Twigg et al. 2013, Glass et al. 2019 and the present study

Family/Patient	Sex	Age at assessment (years)	Phenotype, CS^**a**^	Chari-1 malfomation	Facial dysmorphism	Other phenotypic traits	Reference
family 12	M^b^	4	pansynostosis	unknown	hypertelorism, dysplastic ears	brachydactyly of hands and feet, broad halluces, delayed development, poor attention span, problems with writing	Twigg et al. 2013 [9]
patient 19	M	28	pansynostosis	yes	hypertelorism, malar hypoplasia, frontal bossing, long philtrum, high palate, lowset ears	inverted nipples, clinodactyly	Glass et al. 2019 [12]
patient 25	F^c^	< 1	unicoronal synostosis	no	long philtrum, short turned up nose	joint hypermobility	Glass et al. 2019 [12]
father of patient 25	M	–	–	–	hypertelorism, mild malar hypoplasia, prognathism	–	Glass et al. 2019 [12]
**patient 1 (son/index)**	**M**	**0,16**	**sagittal, bilateral lambdoid**	**yes**	**Constriction of the posterior skull, compensatory forehead expansion, hypertelorism, exorbitism**	**–**	**Baranowska Körberg et al.**
**patient 2 (mother)**	**F**	**4**	**sagittal**	**unknown**	**Hypertelorism, exorbitism**	**poor attention span?**	**Baranowska Körberg et al.**

Abbreviations: ^a^craniocynostosis (CS), ^b^male (M), ^c^female (F)

In patient 25, the variant was paternally inherited and she presented with unicoronal synostosis, long philtrum, and short turned up nose. Interestingly, her father did not present with synostosis, but had orbital hypertelorism, mild malar hypoplasia and prognathism. Both patients had raised ICP, in patient 19 at the age of 28 months, and <  1 month of age in patient 25. This further supports the decreased penetrance and variable expressivity seen in autosomal dominant disorders, between families and within families and even among patients carrying the same variant in the *ERF* gene.

Other heterozygous variants causing CS has previously been reported in *ERF* gene. Twigg et al. sequenced *ERF* in 411 patients and detected mutations in *ERF* in 12 families of which most were truncating variants [9]. There were nine patients (9/12) with multiple synostosis, or pansynostosis, and among them two with BLSS. In 2015, Chaudhry et al. sequenced 40 patients, and heterozygous mutations in *ERF* were detected in 2 patients (5%), one patient with bicoronal and metopic synostosis, and the other with pansynostosis [10]. Glass et al. reported 16 unrelated probands and 20 family members with multi sutural synostosis, predominantly BLSS [12]. In total 13 different heterozygous ERF-variants were reported (Table 1). These studies suggest a wide spectrum of pathogenic variants causing ERF-related CS [9, 10, 12]. The phenotype in the mother and son presented here is consistent with the phenotype previously described.

Hence, our work further supports that *ERF* is a candidate gene that should be considered, particularly in patients presenting with multiple suture or BLSS of CS and in patients with a progressive course.

## Data Availability

The data of the current study are available from the corresponding author on reasonable request.

## Abbreviations

BLSS: Bilateral lambdoid and sagittal synostosis
CS: Craniosynostosis
ERF: ETS2 repressor factor
CT: Computed tomography
MRI: Magnetic resonance imaging
WES: Whole exome sequencing
ICP: Intracranial pressure
